# Oral health-related quality of life in Swedish young adults

**DOI:** 10.3402/qhw.v10.27125

**Published:** 2015-06-09

**Authors:** Gunvi Johansson, Anna-Lena Östberg

**Affiliations:** 1 School of Social and Health Sciences, Halmstad University, Halmstad, Sweden; 2 Department of Behavioural and Community Dentistry, Institution of Odontology, Sahlgrenska Academy, University of Gothenburg, Gothenburg, Sweden; 3 Public Dental Service, Region Västra Götaland, Sweden

**Keywords:** Oral health, quality of life, young adults

## Abstract

The living conditions of young adults in Sweden have changed during the last decades due to the economic and employment situation in society. Although oral health is mainly considered to be good in this age group, their use of dental care has decreased and their priorities and opportunities regarding oral health are little known. The purpose of this study was to describe the views of Swedish young adults on their oral health and oral health-related quality of life (OHRQoL). The design of the study was qualitative, using content analysis. Sixteen young adults, aged 21–29 years, were interviewed. The findings from the interviews were summarized under the theme “Young adults reflected on their OHRQoL in a time perspective” consisting of three categories: “Past experiences, Present situation, and Future prospects.” The OHRQoL of young adults is dependent not only on their own experiences of oral health during childhood and their received dental care but also on their present self-perceived oral health, oral health habits, and social life; together with their expectations of future oral health. The findings in this study indicate that the oral health awareness and needs of young adults, as well as their expectations of oral care, merit further follow-up.

Oral health-related quality of life (OHRQoL) has been defined as an individual's perception of how functional, psychological, and social aspects, together with pain and discomfort, affect personal well-being (Inglehart & Bagramian, 2002). Accordingly, OHRQoL has been described as a multidimensional concept including subjective evaluations of own oral health, as well as expectations of and satisfaction with dental care. Furthermore, Inglehart and Bagramian (2002) suggested that a person's OHRQoL is her or his own assessment of how four different groups of factors affect personal well-being: functional factors, psychological factors, social factors, and the experiences of pain and discomfort. The factors described above are tied to the functioning of the person, the situation, and the interaction between these, which means that an individual's background, past and current experiences of oral health and dental care, as well as views of the future, will influence the response to different situations.

The concept of OHRQoL has also been described as “an integral part of general health and well-being” (Sischo & Broder, 2011). Thus, OHRQoL can be seen as measuring both dental care needs and efficacy of care. Psychosocial factors, such as negative life events involving social readjustment, were found to impact the OHRQoL of young Australian adults (Brennan & Spencer, 2009). Positive aspects, such as optimism, resilience, and coping ability, have likewise been described as having an impact on a person's general quality of life (Broder, 2001; Strauss, 2001).

Oral diseases, specifically dental caries and periodontal disease, are still a major problem worldwide (WHO, 2014). The caries situation has improved during the past decades, but signs of stagnation in young people have been reported in recent years (Haugejorden & Birkeland, 2006; Tanner et al., 2013). Periodontal diseases are less investigated in young people, but Ericsson et al. (2009) found high levels of plaque and gingivitis among 19-year-olds in Sweden, and poor oral hygiene was found especially in male subjects. The prevalence of dental caries as well as periodontal disease varies both between and within countries (Petersen, 2003). In Sweden, epidemiological data on caries in children and adolescents have been available for many years, but not for young adults (National Board of Health and Welfare, 2011). The self-reported oral health is generally good among young people in Sweden and continues to be good but is poorer in socio-economically weak groups (Nordenram, 2012). Another oral problem is dental trauma. The consequences of a dental injury in childhood may persist throughout the individual's life and may, therefore, cause problems in young adulthood (Glendor, 2008). Temporomandibular problems are also of concern. Nilsson, List, and Drangsholt (2005) found in a Swedish study that more than 4% of adolescents (more girls) reported such pain.

In Sweden, dental care is offered through the Public Dental Service and at private clinics. Dental care in Sweden is free of charge for individuals below 20 years of age. Thereafter, an annual subsidy of SEK 300 is offered until the year a person turns 30. A decrease in dental care use has been seen among young adults, especially among men (Nordenram, 2012), for several decades. When young people no longer receive dental care free of charge, there may be a risk that they do not seek dental care until they experience oral problems. Reasons given by young Swedish people for not having regular dental visits were not only strained economy (Johansson & Fridlund, 1996; Östberg, Ericsson, Wennström, & Abrahamsson, 2010; Östberg, Jarkman, Lindblad, & Halling, 2002) but also little perceived need based on good self-rated oral health (Nordenram, 2012). In a Swedish study, 41% of male and 30% of female 19-year-olds were found not to plan for future dental visits when they have to pay for the care (Östberg et al., 2010).

The life situation for young adults in the industrialized world has changed over time during the past 50 years. Their economic situation has become more insecure due to the uncertain labour market, leading to high levels of unemployment and also prolonged education (Arnett, 2007; Lager, Berlin, Heimerson, & Danielsson, 2012; Stone, Berrington, & Falkingham, 2011). Moreover, these circumstances often delay settling into adult roles like marriage and parenthood (Arnett, 2007; Stone et al., 2011). Quite a few young adults still live with their parents, mainly for economic reasons (Hendry & Kloep, 2010; Stone et al., 2011). However, the life situation for young adults differs considerably depending on their employment and/or educational status.

Åström and Wold (2012) followed a cohort of young Norwegian people and found that early sociobehavioural circumstances at age 15 had a great impact on adult oral health at age 30. Oral health awareness was described as poor, in general, among adolescents; and their beliefs in changing their oral health by themselves were limited. In a Swedish study, personal and professional care, social support and impact as well as external aspects like appearance and economy were important for the adolescents’ self-perceived oral health (Östberg et al., 2002). However, less is known about young people's views on their oral health during the transition to adulthood. The aim of this study was, therefore, to describe the views of young adults on their oral health and OHRQoL.

## Design and methodological approach

A qualitative approach was chosen and data collected through qualitative interviews were analysed according to content analysis. Content analysis is a method for the systematic analysis of written, verbal, or visual communication. The method may have an inductive as well as a deductive approach (Krippendorff, 2012). Qualitative content analysis has been defined as a “research method for the subjective interpretation of the content of text data through the systematic classification process of coding and identifying themes or patterns” (Hsieh & Shannon, 2005, p. 1278). The method was chosen as it allows the informants to openly express their experiences and opinions to the researchers. In the analysis, both manifest content and latent content were sought. The manifest content described the visible, obvious components in the text, whereas the latent content dealt with a relationship between different parts of the manifest content and an interpretation of the underlying meaning of the text. Both perspectives demanded interpretation but of different depths and on different levels. This study focused on both manifest and latent content.

### Sampling of informants

The study was performed in south-western Sweden. Strategic sampling according to age, sex, and education was carried out to represent the age cohort of 21–29 years. About half of the invited individuals chose not to participate. The reasons given were often studies or work away from home, but some stated that they were busy or simply “not interested.” Sixteen young adults, eight 21–25-year-olds and eight 26–29-year-olds participated. Of these, nine were females and seven were males. Eight of the informants had either a university degree or were students at a university and eight had completed grammar school. Fourteen received dental care on a regular basis and two were non-attendees. Ten were patients at one Public Dental Service clinic and four of the informants attended one private clinic. A staff member from each clinic and the interviewer selected patients from the clinics’ recall systems. Two non-attendees were recruited from the local university. This sampling was intended to provide data with adequate depth and breadth to fulfil the aim of the study.

### Interview guide

The interview guide covered issues of OHRQoL and the entry questions were: “What is your opinion about your mouth and your teeth? How would you describe quality of life? Can you describe how your mouth and your teeth impact your quality of life? How do you perceive the situation concerning your mouth and teeth in the future?” The informants were encouraged to elaborate on their answers during the interviews. Probing questions were asked, like: “Can you tell me more about that? How did it happen? How did you feel then? Can you give an example? Anything else you want to say?”

### Data collection

The informants were initially contacted by ordinary mail and asked if they were willing to participate in the study. After about 1 week, they were contacted by phone and for those who were interested in participating, an appointment for an interview was arranged. Sixteen open-ended thematic interviews were carried out from June to December 2010 by the first author (GJ), who is an experienced, registered dental hygienist and public health lecturer. The interviews were conducted outside the dental clinics in peaceful environments, such as a parish house or a school office. The current study was the first step in an interview session about OHRQoL measures reported on elsewhere (Johansson, Söderfeldt, & Östberg, 2014).

### Data analysis

The interviews were transcribed verbatim by the interviewer shortly after they were conducted. The data were systematically analysed by both authors. The second author (ALÖ) is a dentist and a researcher. A qualitative content analysis, guided by Graneheim and Lundman (2004), was made. Initially, the interviews were carefully read through several times, line by line, to obtain a sense of the whole and to get an overview of the text before rearranging it into units for analysis. As a first step, meaning units in the text were identified. These meaning units consisted of words or statements with related content and of relevance to the aim. The meaning units were then condensed, which was done by shortening the text but preserving the core content (Graneheim & Lundman, 2004). The condensed meaning units were abstracted and assigned codes. Thereafter, codes with commonality were grouped together into main categories and subcategories. The categories were discussed and reflected upon by the authors and after agreement, the underlying meaning expressing the latent content was formulated into a theme.

### Ethics

The head of the dental clinics allowed access to the patient databases for the selection of informants. Written and verbal information about the aim of the study, assurance of privacy, confidentiality in the presentation of results, and contact information for the responsible author was sent to the intended informants. It was emphasized that participation was voluntary and could be interrupted at any time without a stated reason. The risk of ethical problems may be difficult to predict, as interviews may cause discomfort or negative emotions, although clear information before the interviews may contribute to minimizing those problems. The study was approved by The Regional Ethics Board in Lund (Reg. no.: 2009/124).

## Results

**Figure 1:**
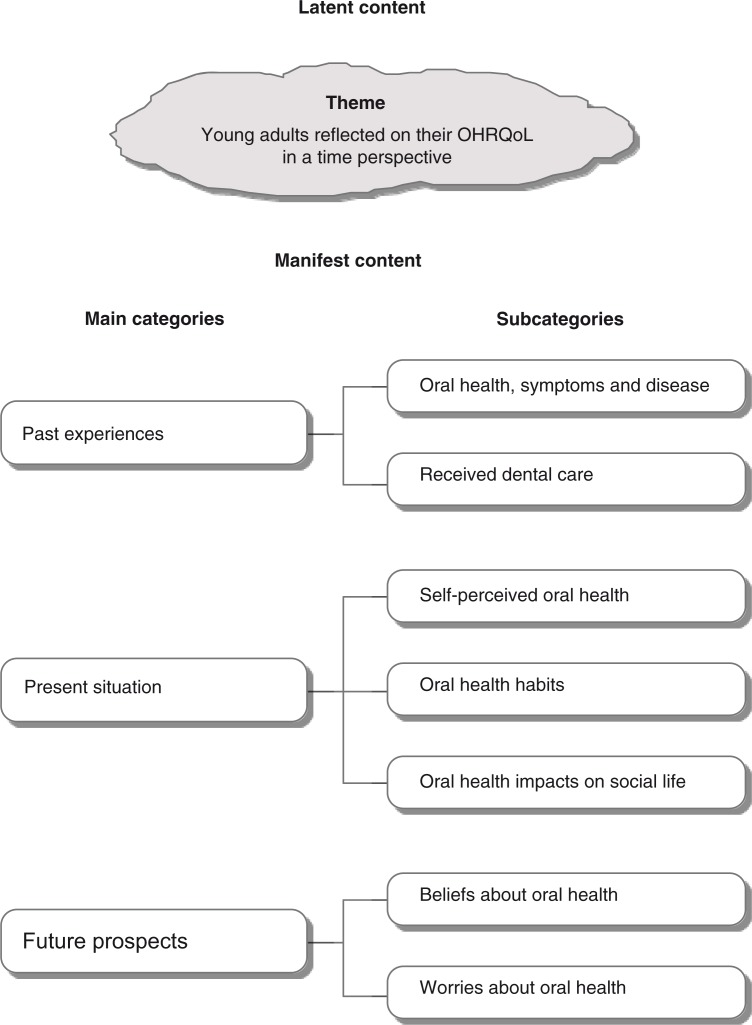
Latent and manifest content with categories and subcategories.

The results were organized in manifest and latent content. Three main categories containing seven subcategories constituted the manifest content (Figure 1). The main categories in the manifest content were “Past experiences, Present situation, and Future prospects.” The latent content was formulated as a theme: “Young adults reflected on their OHRQoL in a time perspective.” The main categories emerged during the analysis, because it became obvious that the young adults’ descriptions of their oral health and their OHRQoL contained a mixture of impressions from their past experiences, their present opinion of their oral situation, and what might happen in their future life. The quotations illustrating the results are chosen to represent all interview protocols.

### Past experiences

The young adults reported that their oral health had mainly been good during their childhood and adolescence but they also reported experiences of different kinds of oral symptoms and disease. Experiences of received dental care were reported as having been both positive and negative. Good relationships with dental staff in the past were described as highly valued and to have an impact on OHRQoL.

#### Oral health, symptoms, and disease

The informants had experienced good oral health during their childhood and adolescence. No experience of oral health problems was described as causing less reflection on their former or present oral health, compared with others of the same age or of older persons. Informants expressed satisfaction with their teeth in the past from different points of view:

Since I haven't really had any immediate problems, I haven't thought about it much, but it's been more of a natural thing for me, but then, at the same time, you think that this is important.

However, the informants reported an array of former oral health problems such as caries, irregular teeth (often corrected by orthodontic treatment), trauma, pain from blisters in the mouth, problems with wisdom teeth, and bruxism. Some informants had no own experience of caries; however, they were aware of the problem and this was frequently brought up, irrespective of their own oral health. Severe caries lesions in early childhood were described as leading to mental stress, numerous dental visits, and a great deal of fuss surrounding their dental health:

I've had cavities in almost every single tooth since I was a child, when I was little… It wasn't as if my teeth bothered me… I can't remember having problems, except that it was a pain to go to the dentist because I always had to have fillings.

Some informants had experiences of orthodontic need and treatment. This was common and often commented on. The need for treatment was reported to be related to irregular teeth, gaps between the teeth, and “teeth growing on top of each other.”

Also, the informants reported dental traumas. Accidents had occurred during sports activities, game-playing, and physical activities, for instance, at school, but also under the influence of alcohol. The consequences differed, from minor injuries, like losing a small part of a tooth, to more serious consequences like losing a whole tooth. However, such minor fillings were often repeatedly fractured or lost and therefore seen as a problem. Some informants reported more severe injuries resulting in prosthetic treatment, such as a crown or an implant. The treatment caused pain and discomfort, but the results were satisfying:

As I said, I got this implant, too, when I was in upper secondary school. I went to the dentist a lot then and had a lot of pain and that wasn't a whole lot of fun… When I was in fifth grade, I was on my bike and fell and had my tooth knocked out.

Some informants reported problems with shooting pain in their teeth, pain or irritation from the gums, and from carious lesions. Impacted wisdom teeth had also caused problems with possible need for surgical removal. This was considered to be an expensive treatment:

…and I've had problems with my wisdom teeth when they came out all awry. They grew all over the place.

Blisters in the mouth or aphthous lesions could be frequent and some females related these to the menstrual cycle. Grinding of teeth (bruxism) was another source of pain, mostly headache, and was described by a few informants. This also had caused fractures of fillings and teeth.

#### Received dental care

The dental care that had been received from the Public Dental Service during childhood was considered as a “good basis,” also in adulthood, for keeping the teeth in a healthy state. Oral problems in childhood were regarded to influence present oral health. For example, severe caries in childhood resulted in a lot of fillings:

Well, you know, I have lots of old fillings and that may not be all that positive later in life… But it's not something that I keep thinking about, because now I have no problems at all.

Contacts with the dental staff during childhood and adolescence were described as both positive and negative. Some of the informants emphasized that a good relationship with the dental staff was very important and sometimes even crucial for the decision to continue with the dental treatment. Dentists and dental hygienists, as well as dental assistants, had given information about oral health and oral hygiene. One informant considered it easier to talk to the dental hygienist or a dental assistant than to talk to a dentist because, “when you see the dentist you only get things done and leave.” Information given about oral hygiene was considered as important and useful in order to attain oral health. There were examples in the data of how health education had influenced informants to better oral health habits. On the other hand, less successful, one-way information was also reported. One informant reported that the dental staff had made clear the importance of flossing aggressively. Strict advice from dental staff to avoid soft drinks was not always successful, as it sometimes led to obstinate behaviour, i.e., increased consumption. However, the informants often expressed satisfaction with the information they had received about “how to take care of the teeth” and to preserve and promote oral health:

When I got a new dentist—she was really angry that I hadn't used dental floss—but after that, my dental hygiene has become much better, too. Now I'm almost addicted to it, 2–3 times a week.

Dental staff and parents seemed to influence the actual oral hygiene habits of young adults. Support from dental staff was considered to be important for oral health habits in adolescence and adulthood. In early childhood, parents seemed to have been important as role models with regard to oral hygiene:

I think that… I grew up with fluoride rinsing at school and that, so I suppose I've grown up with taking good care of my teeth. My parents always made sure that I looked after my teeth, brushed twice a day, and I've used a lot of dental floss and things like that.

The informants were generally satisfied with the reception at the dental clinics. However, some complained that the dental visits could sometimes be unnecessarily time consuming:

Yes, but I don't know if I'll go to the public dental service or privately… because they messed up last time, changed my appointments and that—and then they couldn't do it (remove tartar) at the same time as they examined me.

Orthodontic treatment was considered as important, also by those who had not received that kind of care. One informant had been offered orthodontic treatment, but felt that he was expected to decline the treatment, because of the dental staff's attitude to his needs, and today he regrets the decision. Some informants reported that they wished they had been offered orthodontic treatment during childhood to get straight teeth as adults. Satisfaction with orthodontic treatment was reported, as it could “boost (the person's) confidence” to have straight teeth, and some informants said that they were “grateful for the treatment,” because, without it, “I would not have been so happy.” The reverse situation, where the treatment had failed, was also reported, for example, when retainers were removed prematurely and a relapse occurred:

I had braces before, but then I had that stuff that was on the inside, the steel wire, removed, and then it was a complete mess again. You've had braces all these years and then they say that it's ok and we can take them away now, so I've been annoyed and irritated with that…

Dental treatment, including anaesthetics and drilling, could be painful according to the informants. Injection of a local anaesthetic could entail anxiety and even fear, especially among those having severe problems or trauma treatment:

Well, I had some fillings as a child and they drilled and drilled quite a lot so they hit the nerve and it comes from that, it was a pretty unpleasant experience.

### Present situation

The young adults’ satisfaction with their oral health at present was expressed not only as the absence of caries but also as having an overall positive view of their mouth status. The young adults were aware of the importance of good oral habits for preserving good oral health over time. Uncertainty concerning freshness of the mouth, speaking clearly, and having good-looking teeth was deemed to be important and to impact their social life.

#### Self-perceived oral health

The informants perceived their oral health as being favourable at present and considered themselves as being aware of how to promote oral health and how to avoid risk factors for oral disease. Satisfaction with oral health was expressed in different ways. Absence of caries or new lesions in the teeth seemed to be important. A more comprehensive description was to be satisfied with their teeth and to have a positive view on their teeth and their mouth as a whole.

I think so, I have a good relationship with my mouth—I think we are good friends.

Problems like pain from blisters and shooting pain in the mouth persisted into adulthood. Few informants reported recently diagnosed caries lesions. Still, oral health problems did not always entail worse self-perceived oral health. It could also be a matter of ability to cope with the situation and the state of the mouth, even if it was not regarded as perfect. One informant stated that it was “necessary to accept the situation as it is,” concerning oral conditions.

…you have the mouth you have… it works.

#### Oral health habits

The awareness of good food habits to prevent caries lesions differed. “Sugar addiction” could be noted among the informants and it was thoroughly described how frequent use of sweetened beverages had led to severe pain in a tooth.

I had a wisdom tooth at the back of my mouth that was so incredibly painful that I more or less wanted to kill myself, and then my mother forced me to go to the dentist—she had to drive me—“just get in there.” It turned out that I had drunk so much Coke that the tooth was completely eaten away.

It was felt that dental staff had not always explained possible reasons why some individuals are more affected by caries than others. Some informants thought that they had good knowledge and took good care of their teeth, while others were aware of their poor food habits and poor oral hygiene.

Yes, I changed dentists and when I saw him I had cavities, really big ones in some places, and I don't really know why I'd got them.

Informants were hesitant about whether their oral hygiene was good enough, even if they tried to take care of their teeth. Quite a few reported modified oral hygiene habits over time, either for the better or for the worse. The ambition was mostly to keep up a high level of oral hygiene, but this was considered difficult. Being caries-free was often regarded as “evidence of good oral hygiene.” Some considered that they “should be more careful and use fluoride and dental floss.” Bad conscience could lead to spending a lot of money buying dental oral hygiene equipment (whether used or not):

And I buy all sorts of special toothpaste for a lot of money. Fancy toothbrush, 'cause I imagine that it will get better then. But at the same time I'm doing this because I have a bad conscience about smoking and drinking Coke.

Control over oral health habits was described as a requirement for good oral health. The informants wanted more information from dental staff about food habits and tobacco use, but also general recommendations about how to take care of the teeth. Informants stated that oral health matters or oral hygiene were seldom discussed with their peers.

#### Oral health impacts on social life

Factors impacting social life were fresh breath and the ability to speak clearly. Furthermore, to look good was also considered important for a positive OHRQoL. On the contrary, bad breath or fear of having bad breath was frequently described as causing insecurity and interfering with social situations. One informant using snuff considered this a reason for bad breath, which compromised activities like kissing. On the whole, a fresh mouth was described as being very important:

Mm, what we haven't talked about at all is bad breath. I work as a teacher so I come into contact with a lot of people, and sometimes up close. So that's something I think about quite a bit—and it affects me quite a bit, too.

Concern about how oral health can impact a person's speech was also discussed, even if the informants were uncertain about whether the teeth or something else had caused the problems. “Big teeth” and difficulty of closing the mouth were suggested as possible reasons for articulation problems that could impact encounters with other people:

If my dental health affects my speech, it means that it affects how I'm perceived.

Aesthetics was a frequently stated aspect with an impact on the OHRQoL. Informants expressed great concern with their appearance. The colour of the teeth and whether the teeth are straight and perceived as beautiful was reflected upon. Some mentioned aplasia of permanent teeth; however, this was not a reason for aesthetic concerns.

It's probably important throughout your whole life, but especially when you're young, if you… other things that influence your health, with all the ideals and that, I think it would be a good idea to talk about appearance a bit more.

Moist snuff usage was also regarded as possibly compromising the aesthetics of the teeth, as well as creating gingival retractions. The informants were satisfied with the appearance of their teeth, even if they were not considered to be perfect (whereas others were less satisfied). Orthodontic treatment was discussed as a way to improve the appearance. Another suggestion for getting nice teeth was to bleach them:

Then there's bleaching and that, I used to bleach my teeth… seven or eight years ago. I thought the result was great and it's lasted until almost now.

Aphthous lesions caused pain and were an obstacle to cleaning the teeth, eating, and socializing with others, and could lead to withdrawal behaviour. Thus, it was considered to cause social problems and to impact on quality of life:

… the fact that my mouth hurts means that I don't want to talk because it hurts and I don't want to eat because that hurts, too, and it's obvious that it impacts everything—I can get annoyed with my partner and my sisters and then they can tell straightaway—now her mouth hurts.

The informants often stated that the ability to eat and enjoy food was dependent on oral health and this was considered important for well-being. Pain in the mouth was seen as leading to eating problems, loss of appetite, and consequently, lack of nourishment. It was important to feel comfortable while eating with others and to enjoy the conversation. Taste sensations were also regarded as being of great value.

### Future prospects

The young adults’ beliefs regarding their future oral health ranged from a fatalistic view, where they believed that mere chance determined their future oral health, to a more realistic view, where good oral health habits and regular dental visits were seen as crucial for the outcome. Worries about oral health in the future were associated with dental costs and possible complications after previous oral problems (like caries and trauma).

#### Beliefs about future oral health

As long as the oral health was perceived to be good, the informants did not think about possible future oral problems. The main challenge for the future seemed to be to maintain their present oral health. The hope for the future was that it would stay the same, provided that nothing unexpected happened. Some stated that “oral health problems are something I can deal with when they come.” To take care of your teeth was considered as a way of assuring good short-term oral health, but it was also stated that luck was the reason for good health but that this might change in the future.

Actually, I haven't thought a lot about what will happen to my teeth. I hope they will stay as good as they have so far.

With regard to future expectations, the meaning of good oral health differed a great deal depending on earlier experiences. Some had high demands, including white, straight teeth and no caries, to be satisfied. Others who had received more dental care had lower expectations:

In the future, I will have… good teeth, but I will have to pull some of them out or, yes—remove them and put in a porcelain tooth or whatever they call it.

The planning of future dental visits was considered very important, both by dental attendees and non-attendees. Among the reasons given was the belief that oral problems and large expenses for dental care could be avoided in that way. Although some informants associated dental care with pain and inconvenience and also “something necessarily painful,” this was not generally considered as a reason to refrain from dental visits. However, for some, pain in connection with dental treatment was seen as a cause of dental fear, which could lead to avoiding further dental visits in the long run. The informants considered it risky to avoid dental care, as this could possibly lead to future oral problems.

Actually, I haven't seen a dentist since I got the crown. I think that was 5 years ago. I suppose I have some kind of deep-rooted fear of dentists, and the longer it's been since I last saw a dentist, the more I shy away from going. I suppose I'm a bit worried that there will be a lot of problems with my teeth.

#### Worries about oral health

Worries about future oral health problems, as a consequence of the lack of control of oral hygiene habits, were reported. Informants with present or former caries problems and with old fillings stated that this led to a feeling of uneasiness that was more severe for some informants. For some of the informants, who had experienced severe caries, it had led to a constant awareness of being at risk of new lesions or fillings that might have to be replaced. Severe caries problems in early childhood were discussed by those who had been very caries-active as children. Even if the caries problems had come to an end in adulthood, the memories were reported to remain and lead to insecurity about the informant's oral health situation. However, the uneasiness did not always persist in adult life, if the caries situation was stabilized.

I have some problems every now and again. I've had cavities, for instance, and I feel that that's been bugging me for a long time.

The informants with experience of dental traumas described these as a source of anxiety, as it was difficult to know what would happen in the future with the restorations (filling, crown, or implant)****. Injuries seemed to be associated with insecurity about the future oral health of the informants, irrespective of the extent or the cause of the injury. New injuries might also occur:

Well, since… everything has been rolling along really well for so many years, so I haven't really thought about what to do in the future, but I still play hockey.

There were some worries about how to avoid chewing problems or dentures in the future, despite good self-rated oral hygiene and dental care. Relatives with severe caries problems or other oral problems causing bad oral health led to thoughts about possible heredity. Furthermore, it raised questions about what could be done in addition to good oral hygiene to keep the teeth and the mouth healthy in the long run:

My granny, for instance, she's had cancer of the gums. My granddad had some prosthetic teeth in his mouth—so, of course, I think about it and I think a lot about looking after my teeth now.

The informants mentioned that the cost of dental care was a cause for concern, especially for non-attendees.

It's not cheap to see a dentist—then, on the other hand, I suppose you have to look at it as an investment, to avoid cavities and suchlike before it's too late.

In contrast to those who were anxious to take care of their teeth, it was said that “some other” young people do not feel responsible for their teeth, they “live for the day” and do not “invest” in dental care, because they think it is too expensive. Dental care insurance was discussed as a possibility to avoid high dental costs in the future. However, insurance was hardly seen as an alternative by those who were in great need of dental care because the insurance premium would be too high. The cost of orthodontic treatment in adulthood was perceived as being too high and prevented them from seeking such care.

The informants asserted that if one takes care of the teeth, dental care will be cheap. But if a person gets a caries lesion, the cost of treatment was thought to be extremely high. Only those in gainful employment were considered to be able to afford dental care. It was also a question of priorities; some informants did not want to spend their money on dental care even if they could afford it:

Yes, but right now, it's a question of money—unfortunately, it's like that. Well, you think—yes, it's a lot of money. Then, the fact that I haven't really done anything about it—I suppose laziness comes into the picture—and finding a good dentist—this thing about changing dentists, like, all the time, it's like finding a new hairdresser. I think it's a big bother.

## Discussion

The aim of this study was to describe OHRQoL of young adults. The results of the analysis were summarized in a theme: Young adults reflected on their OHRQoL in a time perspective. Three main categories emerged from the analyses: Past experiences, Present situation, and Future prospects. The category of “Past experiences” contained not only experiences of “oral health, symptoms, and disease” during childhood and adolescence but also views on “received dental care.” The second category, “Present situation,” contained three subcategories: “self-perceived oral health, oral health habits, and oral health impacts on social life.” Two subcategories emerged under the third category, “Future prospects”*:* “beliefs about oral health and worries about oral health.”

In this study, it was shown that the experiences of the young adults’ oral health and dental care were part of their OHRQoL. This finding is in accordance with Inglehart and Bagramian (2002), who suggested that an individual's background, past and current experiences of oral health and dental care, as well as views of the future, will influence the response to different situations. When the informants in our study described their oral health experience, it was often associated with caries or the absence of dental caries. Caries seemed to be of great concern, although many Swedish young adults have no personal experience of the disease. The majority of children and adolescents in Sweden have no caries lesions where fillings are needed (Nordenram, 2012). The informants in our study did not particularly reflect on gingivitis as a consequence of bad oral hygiene, and comments about possible tooth loss in the future were sparse. However, Ericsson et al. (2009) reported poor oral hygiene in a study of 19-year-old Swedes. Experiences of other, more obvious problems, like trauma and aesthetic matters, caused a lot of concern among the informants. Surprisingly, a large number of the informants in this study had experienced trauma. This is in agreement with the findings by Glendor (2009), who described traumatic injuries as an increasing problem that is mainly related to the environment and activity of the individual.

Social support from dental staff and parents emerged in the study as being most important during childhood and adolescence. The importance of social support found in our study confirmed the results from a study of Australian young adults (Brennan & Spencer, 2009), whereas an American study among adults found that lack of financial support, but not social support, reduced OHRQoL (Maida et al., 2012).

The awareness of risk factors for caries seemed to be good in this group, compared with the findings by Östberg et al. (2002), which indicated that oral health awareness among Swedish adolescents was poor. Although the informants in our study had knowledge of the causal connection between oral hygiene, sugar consumption, and caries, they often failed to perform proper daily oral hygiene. Periodically, lack of motivation led to concern and, sometimes, to bad conscience. If dental visits are limited to once a year or every second year, the patients have to keep up their level of oral hygiene for quite a long time without feedback, which might lead to poor oral hygiene. Hugoson, Lundgren, Asklöw, and Borgklint (2007) found that an oral hygiene prevention programme for young adults with follow-up every second month was more successful than programmes where the patients visited the dentist for information and instruction less often. Renewed information or more frequent follow-ups than regular check-ups might be useful but costly and difficult to organize. Choo, Delac, and Messer (2001) argued that oral health promotion should be integrated in general public health programmes. Similarly, Watt and Sheiham (2012) concluded that the best way to reduce oral disease in the long run is to integrate oral health promotion in general health improvement strategies. It was suggested by Choo et al. (2001) and Mårtensson, Söderfeldt, Halling, and Renvert (2004) that media campaigns might enhance the awareness of oral health. There is a large commercial market for oral hygiene products, which may sometimes lead to confusion about what to use rather than to better oral hygiene habits.

It was obvious that the young adults considered their appearance as being very important as it was frequently discussed. An attractive smile has also been found to have a psychosocial impact on the individual's life (Van der Geld, Oostervold, Van Heck, & Kuijpers-Jagtman, 2007). On the other hand, German young adults considered malocclusion a factor that compromised their appearance (Klages, Bruckner, & Zentner, 2004). In an American study (Kiyak, 2008), fear of bad breath, blurred speech, concern about appearance and pain were reported to impact young adults’ social life, which is in agreement with what we found in our study. Adolescence and young adulthood is a period of establishing new social contacts and romantic relationships (Hendry & Kloep, 2002). This could probably be one reason why the appearance of the orofacial area is of great concern to young adults. In a Swedish population survey, one out of four in the age group 20–39 years was dissatisfied with the condition of his/her teeth (Nordenram, 2012). Similarly, minor aesthetic concerns among German university students had significant effects on the perceived OHRQoL, according to Klages et al. (2004).

In this study, the informants believed that three main factors were the most important for oral health: good oral hygiene, avoidance of sugar, and regular visits to a dentist. Regular dental care was also seen as protection against high dental costs in the future. However, dental anxiety and pain in connection with dental treatment were not seen as main obstacles to dental visits. Broadbent, Thomson, and Poulton (2006) concluded that oral health beliefs were associated with dental health behaviour, which was indicated also in our study. Some of our informants had not reflected much on their future oral health but hoped for the best. Similarly, Östberg and Abrahamsson (2013) found that those who believed oral health to be a matter of chance experienced poor self-perceived oral health.

Some informants in the study who had experienced oral problems seemed to have low expectations on their oral health in the future. This is in agreement with what Carr (2001) proposed, namely that expectations are the result of experiences. Thus, poor health could lead to low expectations on health in the future. One way to improve health for people with low expectations would, according to Carr (2001), be to make them aware of the situation and to help them “take control over and improve their own health.”

The uncertain economic situation of young people depends, in part, on the employment situation in Sweden, and the fact that many of them study longer than was common in the past (Arnett, 2007; Hendry & Kloep, 2002). In a study from Switzerland, it was shown that factors contributing to refraining from dental care for economic reasons were associated with lower income, younger age, being female, and current smoking (Peltzer & Pengpid, 2014). A subsidy of SEK 300 per year may not be enough to encourage dental care use among young adults. Being employed was regarded by the informants as a requirement for being able to afford dental care. Young people may have other priorities than dental care (Östberg et al., 2010). One way to reduce the cost of dental care might be to join a capitation plan, where the patients pay a fixed fee for dental care. Furthermore, Johansson et al. (2007) and Andrén Andås, Östberg, Berggren, and Hakeberg (2014) found that patients in a capitation plan received more preventive care than patients in the fee-for-service system.

### Methodological considerations

The design chosen for this study was a qualitative method with the aim to obtain broad as well as deep knowledge of how young adults express their concerns about their OHRQoL. In a qualitative analysis, the reliability should be scrutinized through evaluation of “credibility, dependability, and transferability (Graneheim & Lundman, 2004; Krippendorff, 2012)”.

In our study, credibility was established by transcription of the interviews, shortly after data collection by one of the authors (GJ). The text was read and analysed independently by the two authors. Furthermore, the sample was strategic to get a representative selection of young adults with regard to age, sex, education, and dental attendance as well as dental care regime (PDS or private care). The socioeconomic level was described with reference to education, but other criteria, like ethnicity, might have broadened the scope of the findings. Still, different socioeconomic factors are often correlated (Nordenram, 2012). The informants were interviewed away from the dental clinic in calm and quiet settings, which enhanced the possibilities of relaxed communication. One matter that could possibly have influenced the results was that the informants knew that they were later going to discuss some existing OHRQoL measures (Johansson et al., 2014). This may have increased their awareness of their OHRQoL.

Those declining to participate are, as always, a concern. They may have had divergent perspectives or less interest in the research in question, which might have generated other aspects. The reasons given were plausible, for example, that they had moved to other places for studying or to find a job.

Dependability in a qualitative study measures to what extent the results can be confirmed by others and in a similar context. In this study, dependability was established by consensus between the two authors at the second step of the analysis. Thorough detailed descriptions of the analysis process in combination with quotations from the data can be considered to strengthen the transferability of the study.

## Conclusions

The OHRQoL of young adults is dependent on their experiences of their own oral health in childhood and received dental care, but also on their present self-perceived oral health, oral health habits, and social life, together with their expectations of their future oral health. The findings in this study indicate that the oral health awareness and needs of young adults, as well as their expectations of oral care, merit further follow-up.
